# The clinical characteristics of patients with free perforation in Korean Crohn’s disease: results from the CONNECT study

**DOI:** 10.1186/s12876-015-0262-x

**Published:** 2015-03-18

**Authors:** Young Seok Doh, You Sun Kim, Song I Bae, Jong Pil Im, Jae Hee Cheon, Byong Duk Ye, Ji Won Kim, Young Sook Park, Ji Hyun Lee, Young-Ho Kim, Joo Sung Kim, Dong Soo Han, Won Ho Kim

**Affiliations:** 1 Department of Internal Medicine, Seoul Paik Hospital, Inje University College of Medicine, 85 Geo-dong 2 ga, Jung-gu, Seoul, 100-032 South Korea; 2 Department of Internal Medicine, Seoul National University College of Medicine, Seoul, South Korea; 3 Department of Internal Medicine, Yonsei University College of Medicine, Seoul, South Korea; 4 Department of Internal Medicine, University of Ulsan College of Medicine, Seoul, South Korea; 5 Department of Internal Medicine, Eulji University College of Medicine, Seoul, South Korea; 6 Department of Internal Medicine, Seoul Song Do Colorectal Hospital, Seoul, South Korea; 7 Department of Internal Medicine, Sungkyunkwan University School of Medicine, Seoul, South Korea; 8 Department of Internal Medicine, Hanyang University College of Medicine, Guri, South Korea

**Keywords:** Crohn’s disease, Intestinal perforation, Risk factors, Delayed diagnosis, Cohort study

## Abstract

**Background:**

Free perforation is the most severe and debilitating complication associated with Crohn’s disease (CD), and it usually requires emergency surgery. The aim of this study was to evaluate the incidence of free perforation among Korean patients with CD.

**Methods:**

The CrOhn's disease cliNical NEtwork and CohorT (CONNECT) study was conducted nationwide in Korea, and patients who were diagnosed with CD between 1982 and 2008 were included in this retrospective study. We investigated the incidence of free perforation among these patients and their clinical characteristics.

**Results:**

A total of 1346 patients were analyzed and 88 patients (6.5%) were identified with free perforation in CD. The mean age of the free perforation group was 31.8 ± 13.0 years, which was significantly higher than that of the non-perforated group (27.5 ± 12.1 years) (p = 0.004). Free perforation was the presenting sign of CD in 46 patients (52%). Of the 94 perforations that were present in 88 patients, 81 involved the ileum. Multivariate logistic regression analysis determined that free perforation was significantly associated with being aged ≥ 30 years at diagnosis (OR 2.082, p = 0.002) and bowel strictures (OR 1.982, p = 0.004). The mortality rate in the free perforation group was significantly higher (4.5%) than that in the non-perforated group (0.6%) (p < 0.001).

**Conclusion:**

The incidence of free perforation in Korean patients with CD was 6.5%. Being aged ≥ 30 years at CD diagnosis and bowel strictures were significant risk factors associated with free perforation.

## Background

Crohn’s disease (CD) is a chronic and relapsing inflammatory bowel disease (IBD) that can affect the entire gastrointestinal (GI) tract. The early and accurate diagnosis of CD can be challenging, particularly in patients in whom there is mild clinical activity that mimics symptoms associated with functional GI disorders [1].

Free perforation is relatively rare among the major complications of CD that include stenoses, fistulae and perforations. However, free perforation is the most severe and debilitating complication, and it usually requires emergency surgery. While the incidence of CD remains lower than that in western countries, the recent increases in its incidence in Asian countries are remarkable [2,3]. The characteristics of CD have been found to differ between western countries and Asian countries with respect to its prevalence between gender, the locations of the involved segments, and the frequencies of GI complications [2,4-7]. A higher incidence of free perforation has been reported in Japan (6.8%) [8] compared with western countries (1–3%) [9-13]. However, no large-scale studies have been conducted to investigate free perforation in patients with CD in Korea. The aim of this study is to determine the incidence of free perforation, the clinical characteristics of CD patients with free perforation, and the risk factors associated with free perforation in patients with CD in Korea.

## Methods

### Study population

A total of 1382 patients who had been diagnosed with CD between July 1982 and December 2008 were included in this retrospective cohort of the CrOhn's disease cliNical NEtwork and CohorT (CONNECT) study [14]. Although the CONNECT study could not include all CD patients in Korea, however, 34 institutes were participated in this nationwide cohort study, therefore, we suggested that the CONNECT study could be representative of the Korean CD population. We conducted a comparative study between patients with free perforation and those without free perforation. The study was approved by the Institutional Ethics Review Board of Seoul Paik Hospital.

### Disease diagnosis and classifications

CD was diagnosed according to the diagnostic guidelines for CD in Korea [15]. The diagnoses were systematically re-evaluated before the patients were enrolled in the study and they were confirmed at the end of the study. The final diagnoses were documented in accordance with the disease phenotypes within the Montreal classification [16].

### Spontaneous free perforation

Free perforation in CD was defined as a spontaneous perforation of the small or large bowel accompanied by the flow of the intestinal contents into the peritoneal cavity. We excluded abscess ruptures and secondary perforations that were attributable to other primary lesions and were identified from the patients’ surgical and medical records.

### Clinical characteristics of the patients with free perforation and its incidence

We examined the clinical characteristics of the patients with free perforation in CD, including the time of symptom onset, the year of diagnosis, the therapeutic drug history, and the Montreal classification. We also investigated the anatomical areas in which free perforation occurred and the risk factors associated with free perforation in patients with CD. To evaluate the clinical outcome, we compared the groups in relation to GI complications that often require surgery, hospitalizations, and result in mortality.

### Statistical analysis

The continuous variables are described as the medians and the standard deviations (SD), and the categorical variables are presented as proportions and percentages. The differences between the groups were assessed using the *χ*2 test for categorical variables and independent *t*-test for continuous variables. Multivariate logistic regression analysis was used to examine the associations between the clinical characteristics of the patients and free perforation in patients with CD. Relative risk (RR) was used to evaluate the mortality rate of patients with free perforation. The data were analyzed using PASW Statistics for Windows, Version 18.0 (IBM Corporation, Armonk, NY, USA).

## Results

### Incidence of free perforation

A total of 124 patients were recorded as having any type of perforation associated with CD. Of these, 14 patients were excluded from the study, because thorough evaluations of their clinical and surgical findings determined that their perforations did not satisfy the definition of spontaneous free perforation (8 patients had abscess ruptures, 2 patients had fistulae that mimicked perforations, 2 patients had peptic ulcer perforations, 1 patient had a perforation caused by colonoscopy, and 1 patient had a perforation that was secondary to a GI malignancy). In addition, 22 patients who had suspected but not confirmed free perforations, according to their surgical records, were excluded from the study. Hence, of 1346 patients, 88 (6.5%) were identified with free perforation in CD. Among the 88 patients with free perforation, it was the presenting sign that was associated with a diagnosis of CD in 46 patients (52%), and free perforation developed after the diagnosis of CD in 42 patients (48%).

### Clinical characteristics of CD patients with free perforation

We divided the patients with CD into the free perforation group (n = 88) and the non-perforated group (n = 1258). The mean follow-up periods were 125.9 ± 64.4 months for the free perforation group and 103.7 ± 50.9 months for the non-perforated group. The mean age of the patients in the free perforation group was 31.8 ± 13.0 years, and this was significantly higher than that in the non-perforated group, which was 27.5 ± 12.1 years (p = 0.004).

The median time interval between the first symptom and a diagnosis of CD was 5.1 (0–276.6) months in the free perforation group and 3.5 (0–412.4) months in non-perforated group, a difference that was not significant (p = 0.074).

The location of the disease at diagnosis showed significant differences between the groups, and small bowel involvement was found more frequently in the free perforation group (p = 0.032). Of the 94 perforations that were present in 88 patients, 81 (86.2%) involved the ileum, 6 (6.4%) involved the jejunum, and 7 (7.9%) involved the colon, and 5 patients had perforations at multiple sites (3 patients had perforations in the colon and ileum, 1 patient had perforations in the jejunum and ileum, and 1 patient had 3 perforations in the ileum). Surgery was performed on all of the patients, and it comprised 1 primary repair and 93 segmental resections. We did not identify any mortality that was attributable to the surgery. Table 1 shows the comparison of characteristics between free perforation group and non-perforated group in our CD cohort.

**Table 1:** **Comparison of characteristics between free perforation group and non-perforated group in patients with Crohn’s disease**

	Patients with free perforation (n = 88)	Patients without free perforation (n = 1258)	*P*-value
Gender (female)	22 (25.0)	380 (29.4)	0.355
Age at diagnosis with CD (years)	31.8 ± 13.0	27.5 ± 12.1	0.004
Age at diagnosis ≥30 yr (%)	39 (44.3)	370 (29.4)	0.003
Time from the first symptom to diagnosis (months)*	5.1 (0–276.6)	3.5 (0–412.4)	0.074
Smoking history	15 (17.0)	189 (15.1)	0.640
Family history of IBD	1 (1.1)	31 (2.5)	0.461
CDAI at diagnosis with CD*	161 (30–450)	175 (3–585)	0.658
Disease Location at diagnosis with CD			
L1 (Terminal ileum) ± L4	25 (28.4)	251 (19.4)	0.032
L2 (Colon) ± L4	6 (6.8)	147 (11.4)	0.176
L3 (Ileocolon) ± L4	55 (62.5)	852 (67.7)	0.482
Disease behavior at diagnosis with CD			
Inflammatory (B1)	36 (40.9)	1060 (84.3)	<0.001
Stricturing (B2)	6 (6.8)	123 (9.5)	0.350
Penetrating (B3)	46 (52.3)	75 (5.8)	<0.001
Intestinal complications during whole disease period			
Bowel stricture	36 (40.9)	318 (25.7)	0.001
Perianal fistula	26 (29.5)	471 (37.5)	0.138
Other fistula	20 (22.7)	184 (14.7)	0.040
Intra-abdominal Abscess	21 (23.9)	148 (11.8)	0.001
Number of CD related hospitalization	4.9 ± 7.2	2.9 ± 4.4	0.020
Mortality	4 (4.5)	8 (0.6)	<0.001

Data are expressed as n (%) or mean ± SD or median (range)*.

CD, Crohn’s disease.

IBD, Inflammatory bowel disease.

CDAI, Crohn’s disease activity index.

### Risk factors associated with free perforation

Univariate analysis of the factors associated with free perforation showed that bowel strictures were significantly more frequent in the free perforation group compared with the non-perforated group (40.9% vs. 25.7%, p = 0.001). Significant differences were apparent between the free perforation group and the non-perforated group in relation to the frequencies of other fistulae (22.7% vs. 14.7%, p = 0.040) and intra-abdominal abscesses (23.9% vs. 11.8%, p = 0.001), but the frequency of perianal fistulae did not differ between the groups (Table 1).

Multivariate logistic regression analysis determined that free perforation was significantly associated with being over 30 years of age when CD was diagnosed (odds ratio [OR] 2.082, 95% confidence interval [CI] 1.311–3.307, p = 0.002) and having bowel strictures (OR 1.982, 95% CI 1.243–3.160, p = 0.004). However, the location of the disease in the ileum (p = 0.194), the presence of other fistulae (p = 0.268), and the presence of intra-abdominal abscesses (p = 0.098) were not significantly associated with free perforation (Table 2).

**Table 2:** **Multivariate logistic regression analysis for risk factors of free perforation in patients with Crohn’s disease**

Parameter	OR	95% CI	*P*-value
Age at diagnosis ≥ 30 yr	2.082	1.311-3.307	0.002
Bowel stricture	1.982	1.243-3.160	0.004
L1 (Terminal ileum) ± L4	1.532	0.916-2.562	0.194
Other fistula	1.414	0.767-2.608	0.268
Intra-abdominal Abscess	1.713	0.906-3.239	0.098

### Prognosis of CD patients with free perforation

Twelve patients died during the study, and the mortality of the perforation group (4.6%, 4/88) was significantly higher compared with that of the non-perforated group (0.6%, 8/1258) (RR = 7.72, 95% CI 2.28–26.15, p = 0.001). Eight deaths were attributed to CD, with 3/88 patients (3.4%) died in the perforation group and 5/1258 patients (0.39%) died in the non-perforated group.

## Discussion

It is difficult to accurately determine the incidence of free perforation because of its rarity. The CONNECT retrospective cohort study has enabled us to report that the incidence of free perforation among Korean patients who were diagnosed with CD between 1982 and 2008 is relatively high at 6.5% compared with the incidence of 1–3% in western countries [9-13]. However, our result is similar to the incidence of free perforation in Japanese patients with CD that was reported from a Japanese meta-analysis (6.8%) [8]. To the best of our knowledge, this is the first report of the incidence of free perforation in patients with CD using data from a large-scale multicenter cohort study undertaken in East Asia.

It is widely accepted that the IBD phenotypes differ between Asian and Caucasian populations [2,4,5,14]. With regard to the location of free perforation, Greenstein et al. reported that 50.0% of perforations occur in the ileum and 50.0% of perforations occur in the colon [11]. However, in our study we found that ileal perforations had a dramatically higher incidence (86.2%) and that colonic perforations occurred in only 7.4% of the patients. The Japanese study also reported that 80.0% of perforations occur in the ileum, which is similar to our results [8]. These findings may reflect the different characteristics of these patients and the fact that involvement of the small bowel is more frequent in Asia [4]. Although multivariate logistic regression analysis did not determine an association between the location of the disease in the ileum and free perforation, we think that the perforation risk might be associated with diagnostic difficulties that relate to the location of the initial lesion.

Multivariate logistic regression analysis determined that being over 30 years of age when CD was diagnosed was significantly associated with free perforation (OR 2.082, p = 0.002). In the current study cohort, the mean age at which CD was diagnosed was 31.8 years in the free perforation group. These results had led us to consider that the high rate of free perforation in our study might be attributable to diagnostic delays and subsequent treatment delays, which can lead to poor prognoses [1,17,18]. This concept is supported by the finding that the time interval between symptom onset and diagnosis was a little longer in the perforation group compared with the non-perforated group, but this difference was not statistically significant (p = 0.074). In addition, free perforation was the presenting sign that was associated with a diagnosis of CD in 52% of the patients in the perforation group, while it has been reported as 23–30% in other studies [11]. We consider that the high proportion of free perforation as the presenting sign of CD is indicative of failures to achieve early diagnoses of CD that resulted in an increase in the perforation rate among patients with CD.

Reports from previous studies have suggested that steroids increase the risk of free perforation by obscuring clinical signs and by preventing the closure of perforations [9-12,19-21]. Some studies have reported that anti-tumor necrosis factor (TNF) agents are risk factors for free perforation [22,23]. However, our analysis could not accurately determine the influence of anti-TNF agents, because many of the patients with free perforation were diagnosed before infliximab had been introduced into clinical practice in Korea. Recently, Korean retrospective study including 721 subjects reported that the cumulative probabilities of operation (P = 0.905) and reoperation (P = 0.418) showed no differences between the two groups which divided by the introduction of biologic agents (two groups depending on the date of CD diagnosis; 1987 to 2005 vs. 2006 to 2012) [24]. The authors of this study suggested that a considerable proportion (36.8%) of the second group may have been given anti-TNF agents only after developing a significant level of bowel damage. Therefore, a further prospective study with “early use of biologic agents” would be needed to assess accurately the influence of anti-TNF agents on free perforation [25].

The exact mechanism that underlies free perforation in CD is unknown, but 2 hypotheses have been proposed. The first and most plausible hypothesis suggests that an increase in the intraluminal pressure proximal to a stenosis causes a bowel distension to perforate the thin layer overlying a deep ulcer [8-12,21,26]. In this study, we identified that bowel strictures are an independent risk factor associated with free perforation (OR 1.982, p = 0.004). The second hypothesis suggests that in some patients who do not have dilatations and strictures, bowel ischemia may be attributed to the inflammation of the small vessels [27]. We could not investigate the histopathologic findings in detail nor could we evaluate bowel ischemia in the current retrospective study.

In addition to bowel strictures, complications such as fistulae and intra-abdominal abscesses, developed frequently and hospitalizations were more frequent in the perforation group. The mortality rate was higher in the free perforation group (4.5%) compared with the non-perforated group (0.6%) (RR = 7.72, p < 0.001). Given that there were no deaths from surgical complications, we considered that perforation might be indicative of a poor prognosis, because it is an acute and severe complication that requires emergency surgery, and it is an integral part of the natural course of the disease. Similis et al. similarly reported a higher rate of operative recurrences among patients with CD and perforations compared with those who did not have perforations [28]. However, with improvement in surgical procedures [29-31] and the availability of newer medication including biologics, this may lead to the successful treatment of patients [32].

This study is limited by its retrospective design. First, some of the surgical records did not contain complete descriptions of the patients. Second, we could not accurately evaluate the effects of the medications on the perforations or the complications associated with the perforations, because the dates on which the medications were initiated and the timings of the onset of complications were not accurately recorded. We suggest that future studies should be undertaken that use data from a prospective cohort of the CONNECT study [14].

## Conclusion

The incidence of free perforation in Korean patients with CD was 6.5%, and this is higher than the incidence in western countries, which reflects the specific phenotype of Asian patients with CD. The diagnosis of CD at an older age (≥ 30 years) and bowel strictures were significant risk factors associated with free perforation.

## Abbreviations

CD: Crohn’s disease
IBD: Inflammatory bowel disease
GI: Gastrointestinal
CONNECT study: The CrOhn's disease cliNical NEtwork and CohorT study
SD: Standard deviations
TNF: Tumor necrosis factor
